# Intrinsic homogeneous linewidth and broadening mechanisms of excitons in monolayer transition metal dichalcogenides

**DOI:** 10.1038/ncomms9315

**Published:** 2015-09-18

**Authors:** Galan Moody, Chandriker Kavir Dass, Kai Hao, Chang-Hsiao Chen, Lain-Jong Li, Akshay Singh, Kha Tran, Genevieve Clark, Xiaodong Xu, Gunnar Berghäuser, Ermin Malic, Andreas Knorr, Xiaoqin Li

**Affiliations:** 1 Department of Physics and Center for Complex Quantum Systems, University of Texas at Austin, Austin, Texas 78712, USA; 2 Department of Automatic Control Engineering, Feng Chia University, Taichung 40724, Taiwan; 3 Physical Science and Engineering Division, King Abdullah University of Science & Technology (KAUST), Thuwal 23955, Saudi Arabia; 4 Department of Physics, University of Washington, Seattle, Washington 98195, USA; 5 Department of Materials Science and Engineering, University of Washington, Seattle, Washington 98195, USA; 6 Institut f. Theoretische Physik, Nitchlineare Optik und Quantenelektronik, Technische Universität Berlin, 10623 Berlin, Germany; 7 Department of Applied Physics, Chalmers University of Technology, Gothenburg, Sweden

## Abstract

The band-edge optical response of transition metal dichalcogenides, an emerging class of atomically thin semiconductors, is dominated by tightly bound excitons localized at the corners of the Brillouin zone (valley excitons). A fundamental yet unknown property of valley excitons in these materials is the intrinsic homogeneous linewidth, which reflects irreversible quantum dissipation arising from system (exciton) and bath (vacuum and other quasiparticles) interactions and determines the timescale during which excitons can be coherently manipulated. Here we use optical two-dimensional Fourier transform spectroscopy to measure the exciton homogeneous linewidth in monolayer tungsten diselenide (WSe_2_). The homogeneous linewidth is found to be nearly two orders of magnitude narrower than the inhomogeneous width at low temperatures. We evaluate quantitatively the role of exciton–exciton and exciton–phonon interactions and population relaxation as linewidth broadening mechanisms. The key insights reported here—strong many-body effects and intrinsically rapid radiative recombination—are expected to be ubiquitous in atomically thin semiconductors.

## Abstract (web summary)

The band-edge optical response of transition metal dichalcogenides is dominated by tightly bound valley excitons. Here, the authors use optical two-dimensional Fourier transform spectroscopy to determine the exciton homogeneous linewidth in monolayer tungsten diselenide.

## Graphical abstract

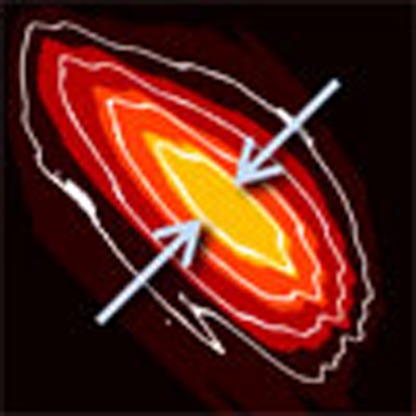

While bulk transition metal dichalcogenides (TMDs) have been investigated over a few decades, recent advances in isolation of atomically thin layers have opened a new regime of semiconductor physics at the ultimate two-dimensional (2D) limit^1^,^2^,^3^. Unlike conventional direct-gap semiconductors such as GaAs, excitons in TMDs form at the *K* and *K'* momentum valleys at the Brillouin zone boundaries with wave functions primarily composed of atomic *d*-orbitals^4^. The exciton has exceptionally large binding energy^5^,^6^ with a non-hydrogenic exciton Rydberg series^6^,^7^, which leads to a small Bohr radius (∼1 nm) in real space and considerable spread in momentum space. Excitons in monolayer TMDs exhibit robust electronic and valley coherence^8^,^9^ as well as coupled spin and valley pseudospin degrees of freedom^1^,^2^ arising from strong spin-orbit coupling and time-reversal symmetry^9^. While these seminal experiments have demonstrated exciting new properties of TMDs and their potential for novel optoelectronic devices, much remains to be learned about the unique exciton physics in these materials. In this work, we elucidate the fundamental mechanisms that broaden the exciton homogeneous linewidth, which is a frequency domain representation of the coherent quantum dynamics.

The exciton quantum dynamics are characterized by two fundamental parameters, illustrated in Fig. 1a. The first is the excited state population relaxation rate *Γ* (inversely proportional to the population decay time *T*_1_), arising from both radiative and nonradiative recombination. The second is the dephasing rate *γ* (inversely proportional to the coherence time *T*_2_) of the coherent superposition of the crystal ground (|0〉) and the exciton (|1〉) states, which defines the homogeneous linewidth of an exciton resonance. The homogeneous linewidth is linked to population relaxation through *γ*=*Γ*/2+γ*, where γ* characterizes pure dephasing processes^10^ such as elastic exciton–exciton and exciton–phonon scattering. In principle, *T*_1_ and *T*_2_ establish the fundamental time scales for quantum optoelectronics (for example, lasers) and quantum information processing applications in semiconductors. They can be probed in either the frequency or time domain using linear and nonlinear optical spectroscopies. In practice, however, local potentials arising from defects and impurities shift the exciton energy and result in an inhomogeneous distribution of exciton frequencies *ω*_*0*_ (Fig. 1b). Inhomogeneity has concealed the intrinsic exciton homogeneous linewidth in all prior low-temperature optical spectroscopy experiments performed on monolayer TMDs.

Here we use optical 2D Fourier transform spectroscopy (2DFTS) to unambiguously separate homogeneous and inhomogeneous broadening of exciton resonances in monolayer WSe_2_. Excitation density and temperature dependent measurements of the homogeneous linewidth reveal an order of magnitude stronger exciton–exciton and acoustic phonon mediated dephasing compared with conventional semiconductors. When extrapolated to zero exciton density and temperature, the residual homogeneous linewidth is ∼1.6 meV, equivalent to a coherence time *T*_2_=0.4 ps. This coherence time is only limited by population relaxation and places a lower bound of *∼*0.2 ps on the radiative lifetime. Interestingly, microscopic calculations predict a similar exciton radiative lifetime for a perfect monolayer WSe_2_ crystal, suggesting that all decoherence mechanisms compete on a sub-picosecond timescale in samples with minimal defects. Such dephasing and relaxation dynamics deviate drastically from those found in conventional semiconductors^11^, where radiative recombination is considerably slower than pure dephasing processes. The new insights obtained by quantifying the exciton resonance broadening mechanisms reported here may provide essential information to guide the extensive efforts developing TMD-based optoelectronics, valleytronics and quantum information devices^12^. For example, coupling TMDs to photonic cavities^13^ may enable novel devices such as exciton-polariton spin switches^14^ and flexible semiconductor lasers^15^. Characterization of the exciton linewidth and dephasing mechanisms would facilitate optimal cavity designs for optical mode matching and efficient light emission.

## Results

### Optical properties of monolayer WSe_2_

**Figure 1:**
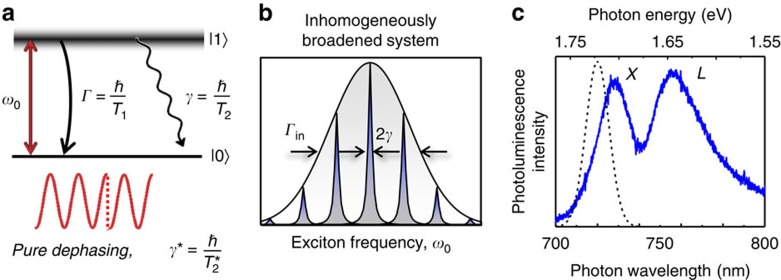
Intrinsic exciton coherent dynamics and resonance broadening mechanisms. (**a**) The quantum dynamics of an exciton with resonance frequency *ω*_0_ are characterized by two key parameters: the population decay rate *Γ* (population lifetime *T*_1_), and the dephasing rate *γ* (coherence time *T*_2_), which defines the exciton homogeneous linewidth. The two are related through the expression *γ*=*Γ*/2+γ*. γ* is the pure dephasing rate describing processes that interrupt phase coherence between the two electronic states without energy loss. (**b**) An inhomogeneous distribution of exciton oscillator frequencies (*Γ*_in_) due to a varying local potential landscape masks the intrinsic homogeneous linewidth in most optical spectroscopy experiments. (**c**) Low temperature (10 K) photoluminescence spectrum (solid curve) features two peaks corresponding to the *A* exciton (*X*) and defect-bound excitons (*L*) at 730 and 760 nm, respectively. The excitation laser used for the nonlinear spectroscopy measurements is shown by the dashed curve.

We examined monolayer WSe_2_ flakes ∼10 μm in lateral size grown on a sapphire substrate using chemical vapour deposition^16^ (Supplementary Fig. 1 and Supplementary Note 1). In monolayer TMDs optical selection rules for exciton states at the *K* and *K'* valleys have led to optical control of carrier spin and valley pseudospin degrees of freedom^8^,^17^. Here we focus on the optical properties of the lowest energy transition corresponding to the *A* exciton of one helicity. The exciton resonance is first identified from the photoluminescence spectrum at ∼10 K shown in Fig. 1c by the solid curve. The spectrum features two peaks—one corresponding to the exciton (*X*) at ∼1,700 meV and the other from defect-bound excitons (*L*) at ∼1,650 meV (Supplementary Fig. 1 and Supplementary Note 1 for details). We estimate the quantum yield to be less than 10% for this sample. The full-width at half-maximum of the exciton peak (*Γ*_in_≈50 meV) is determined by the inhomogeneous broadening as confirmed by the coherent nonlinear spectroscopy experiments presented below. Inhomogeneity can be ascribed to disorder potentials arising from chalcogenide vacancies and other impurities as well as possible excitation of more than one monolayer flake within the laser spot size (∼30 μm diameter).

**Figure 2:**
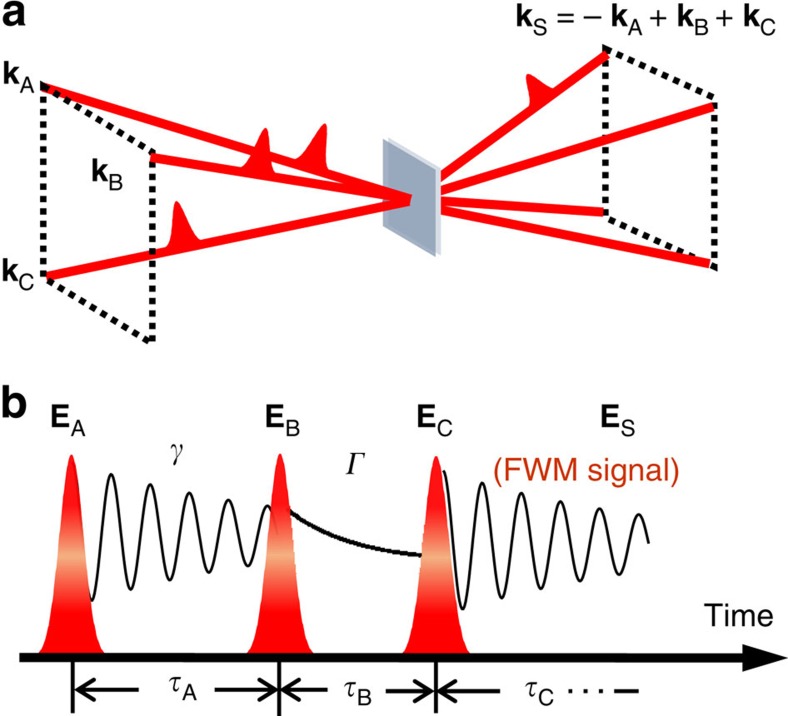
Coherent spectroscopy technique. (**a**) Three phase-stabilized pulses with wavevectors **k**_A_, **k**_B_, and **k**_C_ coherently interact with the sample to generate a photon echo signal that is radiated in transmission in the wavevector phase-matching direction, **k**_S_. (**b**) The emitted photon echo signal field **E**_S_ is measured through spectral interferometry with a phase-stabilized reference pulse as delay *τ*_A_ or *τ*_B_ is scanned with interferometric precision. Exciton coherent dynamics (*γ*) are revealed by scanning the delay *τ*_A_ while holding the delay *τ*_B_ fixed, whereas incoherent population dynamics (*Γ*) are measured by scanning *τ*_B_ with *τ*_A_ fixed.

**Figure 3:**
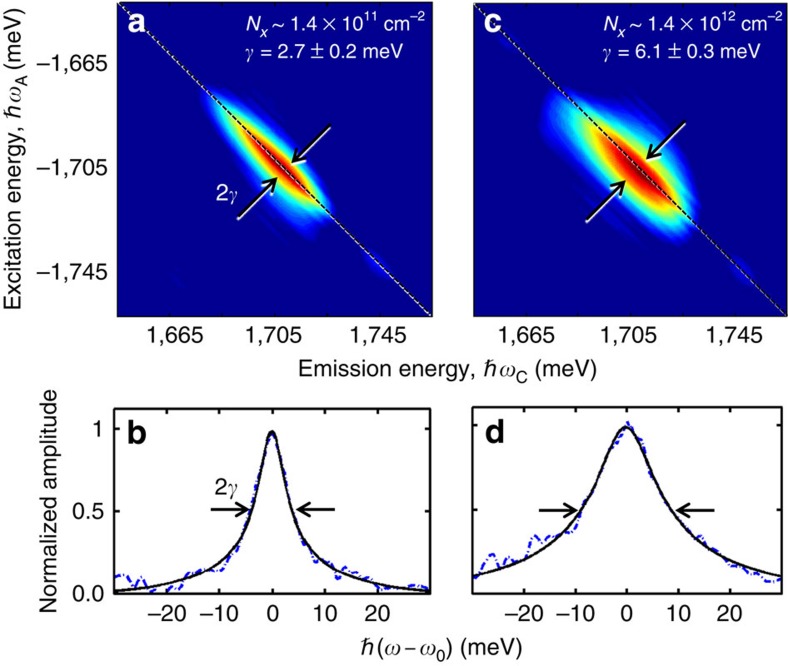
2D Fourier-transform spectra of the bright valley exciton. (**a**) The photon echo signal appears as a single peak in the normalized 2D spectrum (absolute value), acquired using co-circularly polarized pulses and an exciton excitation density of *N*_*X*_∼1.4 × 10^11^ cm^−2^. The peak is inhomogeneously broadened along the diagonal line connecting *ℏω*_A_=−*ℏω*_C_, whereas the half-width at half-maximum of the cross-diagonal lineshape provides a measure of the homogeneous linewidth, *γ*=*ℏ/T*_2_ (indicated by the arrows). A normalized homogeneous profile relative to the exciton resonance frequency, *ω*_0_, is shown in (**b**). The half-width at half-maximum of a square root of Lorentzian fit function yields *γ*=2.7±0.2 meV. (**c**) A 2D spectrum for an increased exciton excitation density of *N*_*X*_∼1.4 × 10^12^ cm^−2^. (**d**) The corresponding lineshape yields *γ*=6.1±0.3 meV. Error bars are estimated by the s.d. from multiple measurements at each excitation density.

To extract the homogeneous linewidth from an inhomogeneously broadened system, we use optical 2DFTS, which is a phase-resolved, three-pulse photon echo (four-wave mixing) experiment with interferometric stabilization of the pulse delays (Methods, Supplementary Fig. 2, and Supplementary Note 2)^18^. We resonantly excite the inhomogeneously broadened excitons using a sequence of three phase-stabilized laser pulses separated by delays *τ*_A_ and *τ*_B_, shown in the schematic in Fig. 2. The coherent light–matter interaction generates a photon echo signal field, **E**_S_(*τ*_A_, *τ*_β_, *τ*_C_), that is emitted during a third time *τ*_C_. The field amplitude is detected through spectral interferometry with a fourth phase-stabilized reference pulse while stepping the delay *τ*_A_. Numerical Fourier transformation of the signal with respect to *τ*_A_ yields a 2D coherent spectrum, **E**_S_(*ℏω*_A_, *t*_B_, *ℏω*_C_), that correlates the excitation and emission energies of the exciton. The absolute value of **E**_S_ is shown in Fig. 3a for co-circular polarization for all pulses, a sample temperature of ∼10 K, and an exciton population density of *N*_*X*_=1.4 × 10^11^ cm^−2^, which is calculated using the laser pulse properties and material absorbance (Supplementary Note 2). The *ℏω*_A_ axis is plotted as negative energy because the system evolves during *τ*_A_ with the opposite phase accumulation relative to that during the detection time *τ*_C_—a result of the photon echo time-ordering of the pulses (Supplementary Fig. 2 and Supplementary Note 2). The spectrum features a single peak on the diagonal line along *ℏω*_*3*_=−*ℏω*_*1*_ indicating the system coherently evolves with the same frequency during *τ*_Α_ and *τ*_C_.

Inhomogeneous broadening of the exciton resonance appears as a continuous elongation along the diagonal dashed line in Fig. 3a. In the present experiments the diagonal linewidth is limited by the laser bandwidth and does not reflect the amount of inhomogeneity as determined from the photoluminescence spectrum. In contrast, the intrinsic homogeneous linewidth of an individual exciton resonance is manifest as the width of the cross-diagonal lineshape along *ℏω*_C_=*ℏω*_Α_, which is shown as the dashed line in Fig. 3b for an exciton resonance at 1,710 meV. In the limit of strong inhomogeneity as seen here, the homogeneous lineshape is well-described^19^ by the square root of a Lorentzian function with a full-width at half-maximum equal to 2*γ*. A least-squares fit to the data yields *γ*=2.7±0.2 meV corresponding to an exciton coherence time *T*_2_=*ℏ*/*γ*=250±20 fs. The linewidth extracted from the 2D spectrum is in excellent agreement with the time-domain decay rate for an inhomogeneously broadened system, verifying the photon echo nature of the nonlinear signal (Supplementary Fig. 2 and Supplementary Note 2).

### Excitation-induced dephasing

**Figure 4:**
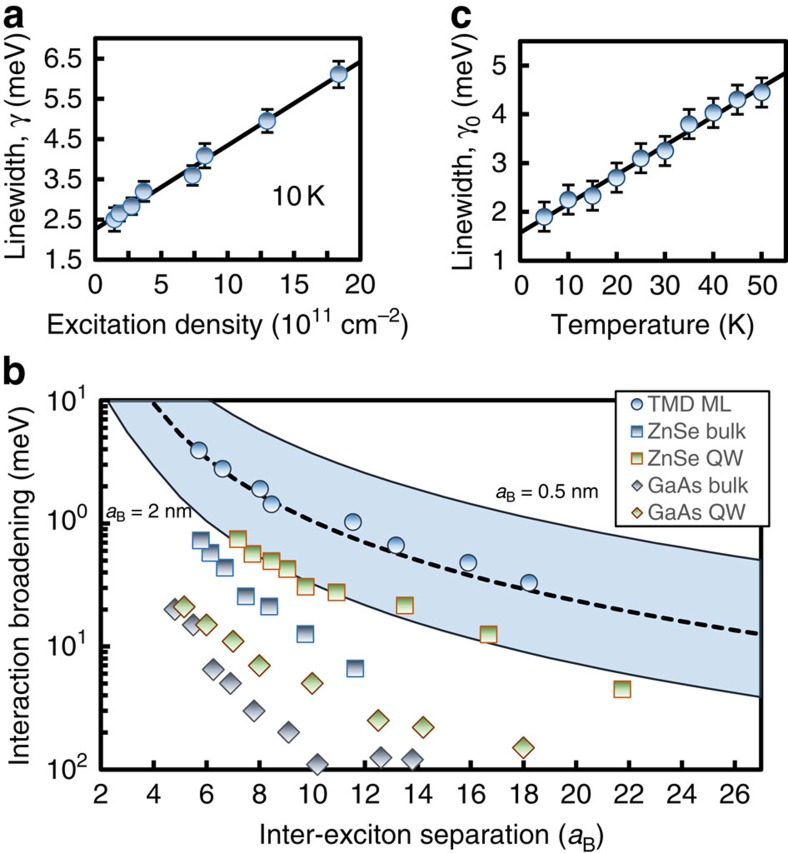
Homogeneous linewidth broadening due to exciton-exciton and exciton-phonon interactions. (**a**) The exciton homogeneous linewidth or dephasing rate (points) for exciton excitation densities ranging from *N*_*X*_∼1 × 10^11^ to ∼1.4 × 10^12^ cm^−2^ at 10 K. The linewidth increases linearly with density as expected for excitation-induced dephasing arising from exciton–exciton interactions. (**b**) Dependence of exciton–exciton interaction broadening on the inter-exciton separation distance in units of the respective Bohr radius for each system. The shaded region is the estimated exciton separation distance in monolayer TMDs for Bohr radii in the range of 0.5 nm<*a*_B_<2 nm, and the solid line is for *a*_B_=1 nm. The GaAs and ZnSe data were taken from (refs 22), respectively. (**c**) The extrapolated zero-excitation density linewidth *γ*_0_ increases linearly with temperature at a rate *γ′*=60 μeV K^−1^. The residual zero-density, zero-temperature linewidth is *γ*_0_(0)=1.6±0.3 meV.

Compared with previous studies that have only probed incoherent population dynamics (*T*_1_ processes) in TMDs, measurements of the exciton coherence (*T*_2_ processes) are particularly sensitive to many-body interactions, which perturb the phase evolution of the coherent signal. We present in Fig. 3c,d a 2D spectrum and homogeneous lineshape, respectively, for an increased excitation density of *N*_*X*_=1.4 × 10^12^ cm^−2^. We confirmed that all experiments up to the highest power are performed in the *χ*^(3)^ regime. For example, the signal field amplitude varies proportionally to the product of the three incident fields. We find that with increasing excitation density, the homogeneous linewidth, or dephasing rate, increases by more than a factor of two—a clear signature of excitation-induced dephasing (EID)^20^ (Fig. 4a).

We now discuss possible mechanisms that contribute to the EID effect observed. First, resonant excitation of the exciton resonance rules out exciton–free carrier interaction as the main linewidth broadening mechanism, since only the exciton density is directly changed with varying laser power. Second, charged excitons (trions) are known to have a binding energy of ∼30 meV in monolayer WSe_2_ and may be masked under the broad exciton inhomogeneous linewidth in the photoluminescence spectrum. However, their contribution to the nonlinear signal is minimized because the trion binding energy is large compared to the excitation laser bandwidth and the laser is tuned to the high energy side of the exciton resonance identified in photoluminescence. Thus, it is reasonable to suggest that the EID observed is dominated by exciton–exciton interactions, an assumption made in the discussion below.

Following a similar analysis performed for quasi-2D quantum wells^21^, EID can be described by *γ*(*Ν*_*X*_)=*γ*_0_+*γ*_*X*_*Ν*_*X*_, where *γ*_0_ is the zero-density linewidth and *γ*_*X*_ is an exciton–exciton interaction parameter. A fit to the data (solid line in Fig. 4a) yields an extrapolated *γ*_0_=2.3±0.3 meV (*T*_2_=0.29±0.04 ps) and *γ*_*X*_≈2.7 × 10^−12^ meV cm^2^ for a sample temperature of 10 K. Strong EID suggests that many-body effects dominate the nonlinear optical response in TMDs, similar to conventional semiconductors. To compare the interaction strength between systems of different material composition and dimensionality, it is illustrative to recast the data in Fig. 4a in terms of the inter-exciton spatial separation. We show in Fig. 4b exciton-exciton EID, defined as Δ*γ*≡*γ*(*N*_*X*_)—*γ*(0), as a function of the inter-exciton separation distance *r*_*x*_ normalized to the exciton Bohr radius, *a*_B_. The exciton spacing can be calculated from the excitation density for 2D and three-dimensional systems using 
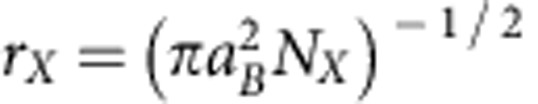
 and 
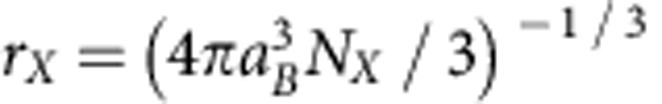
, respectively. The measured EID for monolayer WSe_2_ (solid circles) and a fit to the data (dashed line) are shown for an estimated *a*_B_=1 nm. The shaded region is the calculated Δ*γ* for a range of TMD exciton Bohr radii 0.5 nm≤*a*_B_≤2 nm, where a larger *a*_B_ results in a smaller normalized excitation separation. In monolayer WSe_2_, the exciton–exciton interaction strength is enhanced by an order of magnitude or more compared to conventional semiconductor systems, such as ZnSe (squares) and GaAs (diamonds) bulk and quasi-2D semiconductor quantum wells^22^,^23^. Enhanced interactions in monolayer TMDs can be qualitatively explained by the reduced dielectric screening of the Coulomb force in atomically thin materials^24^.

### Phonon-induced dephasing and population relaxation

We further examine the role of phonons as a resonance broadening mechanism by repeating the excitation-density dependent measurements of *γ* as a function of temperature. We show the extrapolated zero-density homogeneous linewidth *γ*_0_ for temperatures up to 50 K in Fig. 4c. The linewidth increases from 1.9±0.2 meV at 5 K to 4.4±0.3 meV at 50 K (*T*_2_=0.35±0.04–0.15±0.01 ps). This behaviour is reminiscent of exciton dephasing in semiconductor quantum wells due to scattering with an acoustic phonon with energy much smaller than *k*_B_*T*, where *T* is the sample temperature^25^. Single-phonon anti-Stokes scattering can be modelled by *γ*(*T*)=*γ*_0_(0)+*γ′T*, where *γ′* denotes the exciton-phonon coupling strength and *γ*_0_(0) is the residual exciton dephasing rate in the absence of exciton–exciton and exciton–phonon interactions. A fit to the data (solid line in Fig. 4c) yields *γ′*=60 μeV K^−1^, which is a factor of 5–10 larger compared to quasi-2D semiconductor quantum well systems^22^,^23^ and is twice as large compared to bulk TMD InSe in which optical phonons were shown to also contribute to low temperature (<60 K) exciton dephasing^26^. We extract a residual exciton linewidth *γ*_0_(0)=1.6±0.3 meV (*T*_2_=0.41±0.05 ps) at zero temperature.

In order to reveal the remaining resonance broadening mechanisms after eliminating exciton–exciton and exciton–phonon interaction effects, we investigate the population decay by measuring the normalized time-integrated four-wave mixing signal field as a function of delay *τ*_B_ for fixed delay *τ*_A_=0 fs (Supplementary Fig. 3). This experiment is equivalent to the standard pump/probe technique commonly used. The decay follows a bi-exponential function (solid line), yielding fast and slow population decay rates *Γ*_fast_=3.2±0.4 meV and *Γ*_slow_=40±7 μeV. The data are acquired at 5 K and for an exciton excitation density of *N*_*X*_=1.4 × 10^11^ cm^−2^; however, we find that *Γ*_fast_ is independent of excitation density over the range used in the coherent measurements of *γ*. Bi-exponential relaxation dynamics have also been observed in previous ultrafast spectroscopy experiments. The fastest exciton population relaxation rate previously reported is nearly an order of magnitude slower than our extracted *T*_1_ time in both tungsten- and molybdenum-based 2D materials, possibly due to limited temporal resolution^27^,^28^. We attribute the fast decay to population relaxation of bright excitons. The bi-exponential decay can only be explained by additional states (for example, a dark state or a localized state) beyond a simple two-level system. The slow component could arise from decay of energetically degenerate localized excitons or repopulation of the bright exciton from dark states. The slow component could also be partially attributed to an effective lifetime of a thermal distribution of excitons with non-vanishing center-of-mass momentum, which was recently predicted to decay on a few-picosecond timescale^29^,^30^. The fact that the residual homogeneous linewidth is equal to half the population decay rate at low temperature and excitation density is a surprising result, since it suggests that the mechanisms responsible for fast population relaxation do not introduce additional pure dephasing (γ*=0), that is, the homogeneous linewidth is limited by the population decay rate. Pure dephasing processes such as those due to phonon emission are possible at low temperature and excitation density, but they are not observed on the experimental time scales. Exciton–electron and exciton–trion interaction can also contribute to the homogeneous linewidth, in principle. Future experiments performed on gated samples with controlled carrier density and spectrally separated exciton and trion resonances may further clarify their roles in exciton dephasing and population relaxation.

## Discussion

One might expect a narrower homogeneous linewidth or, equivalently, a longer coherence time in a different sample with reduced defect density. However, our microscopic calculations reveal that for completely delocalized excitons in an ideal 2D WSe_2_ crystal, radiative decay becomes the dominant dephasing process due to the large exciton oscillator strength^6^, leading to a residual linewidth of *γ*_0_(0)≈1.43 meV (*T*_2_≈0.5 ps) (Supplementary Fig. 4, Supplementary Fig. 5, and Supplementary Note 3). Fast radiative decay is consistent with our current measurement, which provides a lower bound of ∼0.2 ps on the exciton radiative lifetime. Interestingly, a recent study predicts that as the exciton localization length decreases in samples with more defects, the radiative lifetime increases^31^. Thus, we speculate that as the impurity density in the sample is reduced, the residual linewidth may not experience significant change while the underlying mechanism for quantum decoherence becomes dominated by radiative decay. This speculation is partially validated by similar dephasing times measured from another CVD grown monolayer WSe_2_ sample exhibiting reduced photoluminescence from the defect-bound exciton state (Supplementary Fig. 6).

In summary, we have measured the homogeneous linewidth of excitons and examined various linewidth broadening mechanisms in monolayer WSe_2_. We found that exciton–exciton and exciton–phonon interaction and population recombination to be the dominant mechanisms for quantum dephasing. These processes all compete on a sub-picosecond time scale, which is beyond the temporal resolution of many optical spectroscopy studies performed on TMDs so far. This scenario is drastically different from those found in conventional organic and inorganic semiconductors. For example, in organic semiconductors such as pentacene, the radiative lifetime is typically on the order of nanoseconds while the dephasing time is on a picosecond time scale, making TMDs an attractive alternative for flexible and ultrafast light emitting devices^32^,^33^. Inorganic semiconductor nanostructures, such as GaAs quantum wells, typically exhibit ∼1% absorption, a picosecond long exciton coherence time dominated by pure dephasing processes due to exciton-phonon coupling, and tens of picoseconds radiative lifetime^11^,^34^. The intrinsically rapid radiative decay in TMDs derives from the concentration of oscillator strength at the exciton resonance with exceptionally large ∼10% absorption in a single layer, making strong coupling between exciton and photonic cavity readily observable^13^. These polariton modes may lead to lasers with ultralow threshold or Bose-Einstein condensates^35^.

## Methods

### Sample preparation

Monolayer WSe_2_ was obtained using chemical vapour deposition as described in detail in (ref. 16). The samples were synthesized on a double-side polished sapphire substrate for optical experiments in transmission. The monolayer thickness was verified using atomic force microscopy. The sample was mounted in a liquid helium cold-finger cryostat and kept at a temperature from 5 to 50 K.

### Photoluminescence and 2DFTS experiments

The photoluminescence spectrum was obtained using circularly polarized 532-nm continuous wave excitation. Photoluminescence was collected in transmission with the sample in vacuum. For the nonlinear experiments, 100-fs pulses generated from a mode-locked Ti:sapphire laser at a repetition rate of 80 MHz were split into a set of four phase-stabilized pulses using a set of nested and folded Michelson interferometers. Three of the pulses separated by delays τ_A_ and τ_B_ coherently interact with the sample to generate a nonlinear four-wave mixing signal field **E**_S_(τ_A_, τ_B_, τ_C_) that is emitted during a third time τ_C_ (Fig. 2). The spectral interferogram of **E**_S_ with a fourth phase-stabilized reference pulse is recorded as the delay τ_A_ is scanned while holding τ_B_ equal to zero. 2D Fourier transformation of the spectral interferogram yields a 2D coherent spectrum of the signal field **E**_**S**_(*ħω*_A_, τ_B_=0, *ħω*_C_). Extraction of the signal field is enabled through heterodyning with the reference pulse and phase stabilization up to λ/300 of the pulse delays, which allows the pulses to be phase cycled at each delay for noise suppression. Supplementary Note 1 and Supplementary Note 2 elaborate on the sample preparation method and 2D Fourier-transform spectroscopy set-up, respectively.

## Additional information

**How to cite this article:** Moody, G. *et al*. Intrinsic homogeneous linewidth and broadening mechanisms of excitons in monolayer transition metal dichalcogenides. *Nat. Commun.* 6:8315 doi: 10.1038/ncomms9315 (2015).

## Supplementary Material

Supplementary Information Supplementary Figures 1-6, Supplementary Notes 1-3 and Supplementary References.
