# Supplementary material for: Intrinsic homogeneous linewidth and broadening mechanisms of excitons in monolayer transition metal dichalcogenides
Source: Nat Commun. 2015 Sep 18;6:8315. doi: 10.1038/ncomms9315 (PMC4595717; doi:10.1038/ncomms9315)
Supplement: Supplementary Information — Supplementary Figures 1-6, Supplementary Notes 1-3 and Supplementary References. [file ncomms9315-s1.pdf]

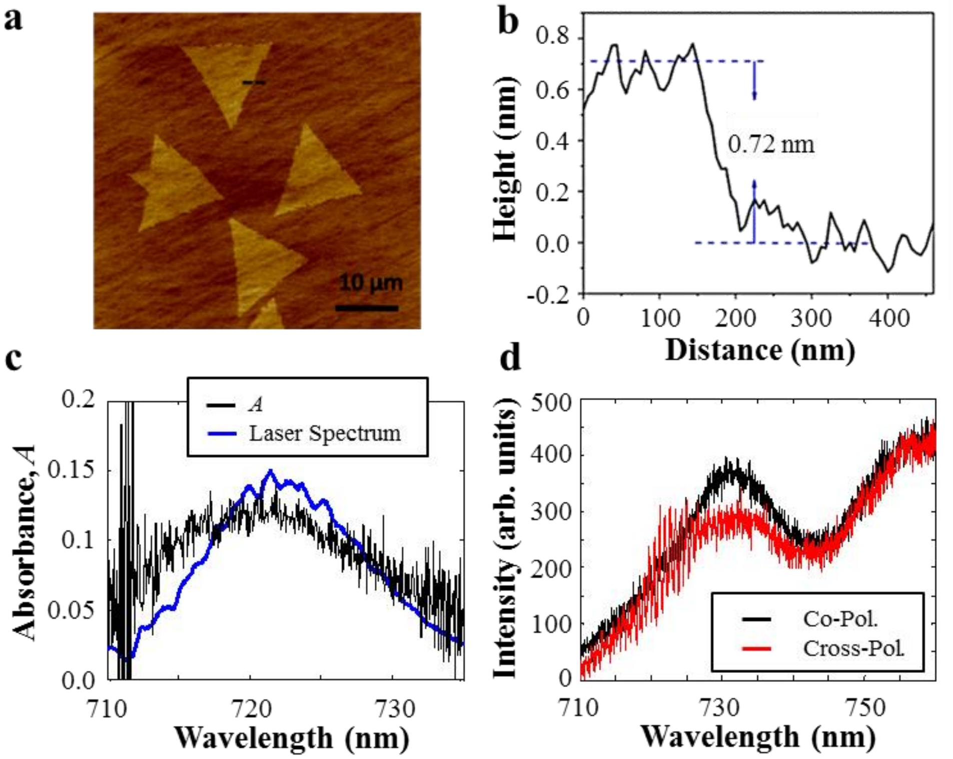

Supplementary Figure 1: Mechanical and optical characterization of monolayer WSe_2. (a) Atomic force microscopy image of single monolayer WSe_2 flakes on a sapphire substrate. **(b)** Height profile along dashed line in **(a)**. **(c)** Absorbance determined from differential reflection measurements at 17 K using the laser spectrum, yielding a maximum $A = 0.12$ at the peak of the laser. The laser spectrum (blue curve) is overlaid for reference. **(d)** Low temperature photoluminescence spectrum for co-linearly (black curve) and cross-linearly (red curve) polarized excitation and detection.

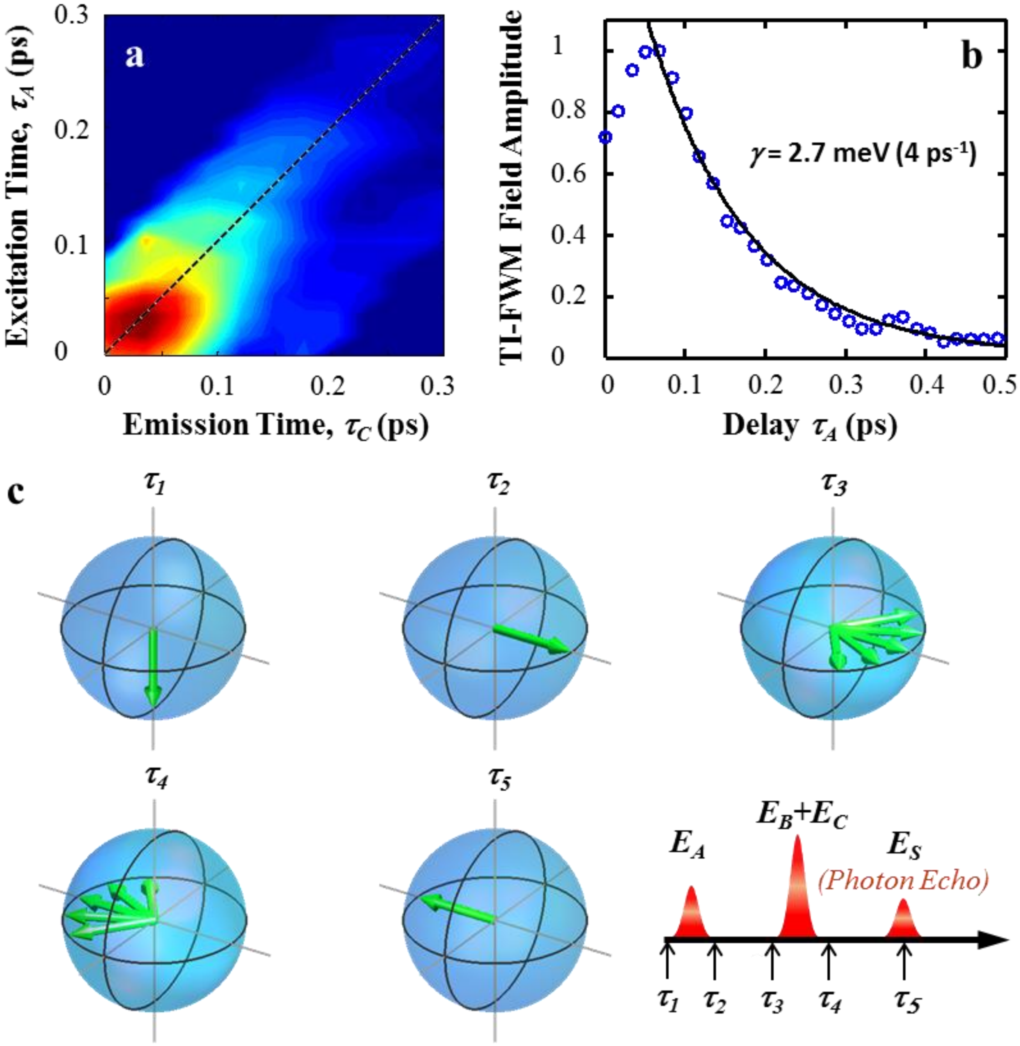

Supplementary Figure 2: Coherent dynamics of valley excitons. (a) A two-dimensional map of the four-wave mixing field during times τ_A and τ_C. The photon echo appears as a single peak on the diagonal dashed line. **(b)** The time-integrated photon echo signal field (points) vs. delay τ_A decays exponentially (solid line) with a rate equal to 2γ, yielding the exciton dephasing rate γ = 2.7 meV (4 ps^{-1}). **(c)** The exciton coherent dynamics are illustrated using the Bloch sphere, which is a geometrical representation of the exciton pure state vector. Synchronous excitation of the inhomogeneously broadened exciton resonance by pulses $\boldsymbol{E_A}$, $\boldsymbol{E_B}$, and $\boldsymbol{E_C}$ generates a signal field $\boldsymbol{E_S}$ that is radiated as a photon echo.

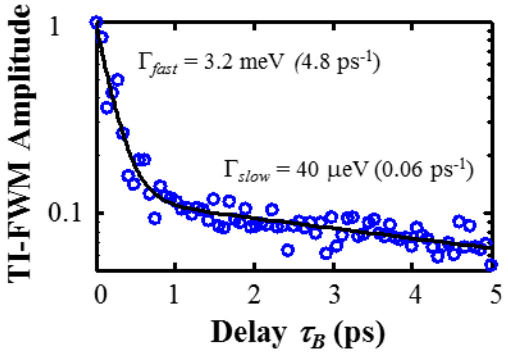

Supplementary Figure 3: Ultrafast exciton population recombination. Exciton population decay measured by integrating the four-wave mixing signal field during τ_C and scanning delay τ_B with τ_A = 0 ps. The ultrafast decay rate Γ_{fast} = 3.2 meV (4.8 ps^{-1}) reflects the intrinsic bright exciton population dynamics.

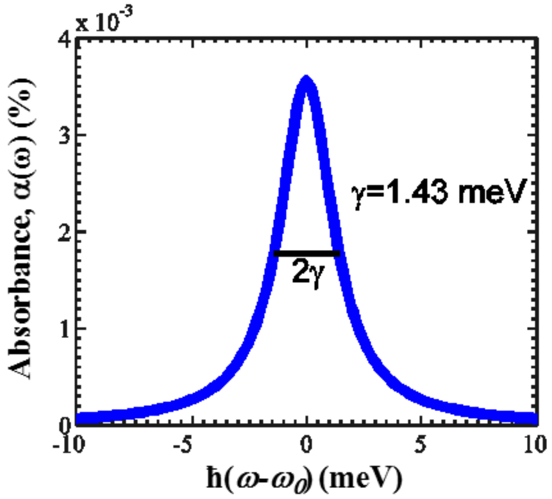

Supplementary Figure 4: Excitonic frequency-dependent homogeneous absorbance α(ω) of WSe_2 exhibiting the delocalized *A* exciton. Our calculations presented in detail in Supplementary Note 3 reveal a lower limit for the homogeneous linewidth of γ = 1.43 meV (2.2 ps^{-1}) due to radiative coupling.

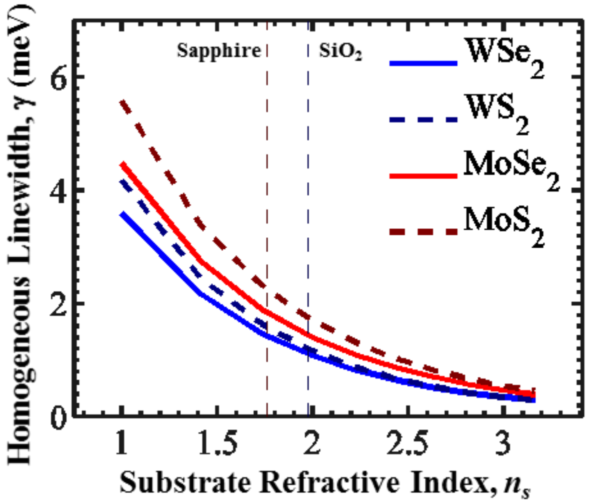

Supplementary Figure 5: Dependence of the exciton homogeneous linewidth on the dielectric environment for monolayer TMDs. The exciton homogeneous linewidth (γ) decreases with increasing substrate refractive index, n_1, resulting in a longer radiative lifetime due to more efficient screening of the Coulomb interaction. TMD materials with a larger effective mass exhibit stronger radiative coupling and a larger intrinsic linewidth.

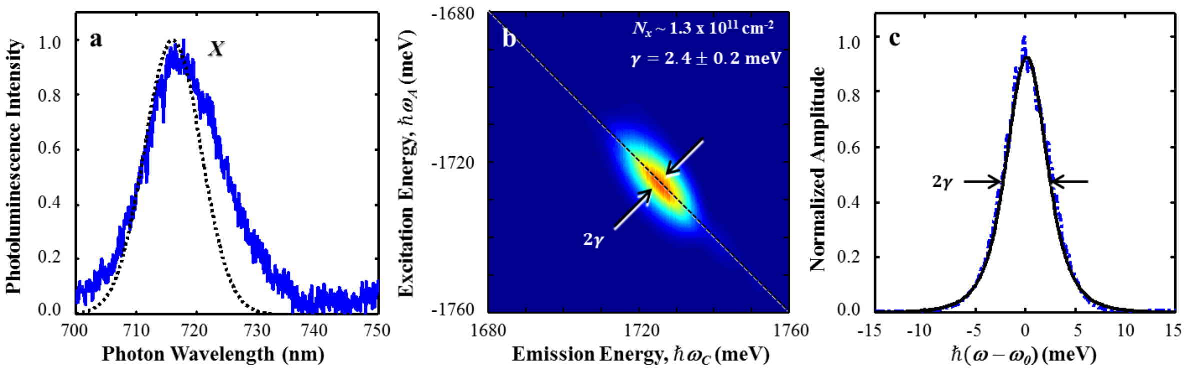

Supplementary Figure 6: Exciton dephasing from monolayer WSe_2 exhibiting reduced defect density. (a) Low temperature (10K) photoluminescence spectrum (solid blue curve) features a single inhomogeneously broadened resonance attributed to the *A* exciton (*X*). The excitation laser used for the nonlinear spectroscopy measurement is shown by the dashed curve. (b) Normalized two-dimensional spectrum for an excitation density of N_X ~1.3×10^{11} cm^{-2}. (c) Normalized homogeneous profile (dashed blue curve) and lineshape fit yielding a homogeneous linewidth γ= 2.4±0.2 meV.

Supplementary Note 1

Growth of monolayer WSe_2: The WSe_2 monolayer film was synthesized using chemical vapor deposition described in detail in Ref. *(1)*. In brief, a double-side polished sapphire (0001) substrate (from *Tera Xtal Technology Corp.*) was cleaned in a H_2SO_4/H_2O_2 (70:30) solution at 100 °C for one hour. The substrate was then placed on a quartz holder in the center of a one inch tubular furnace. WO_3 powder (0.3 grams, 99.5% from *Sigma-Aldrich*) in a ceramic holder was placed in the heating zone center of the furnace and the Se powder (99.5% from *Sigma-Aldrich*) in the tube upstream position and maintained at 270 °C during the reaction. The sapphire substrate for growing WSe_2 was located at the downstream side, where the Se and WO_3 vapors were brought to the targeting sapphire substrates by an Ar/H_2 flowing gas (Ar = 80 sccm, H_2 = 20 sccm, chamber pressure = 3.5 Torr). The center heating zone was heated to 925 °C at a ramping rate of 25 °C min^{-1}. We note that the temperature of the sapphire substrate was at ∼750 to 850 °C when the center heating zone reached 925 °C. The heating zone was kept at 925 °C for 15 min and the furnace was then naturally cooled to room temperature. The reaction yielded triangular shaped flakes of WSe_2 with a base width of ~10 μm. The thickness was measured using atomic force microscopy, with a representative image shown in Supplementary Figure 1a. A height profile along the dashed line is shown in Supplementary Figure 1b, confirming the ~7 Å monolayer thickness of the flakes.

The photoluminescence spectrum shown in Fig. 1c of the main text was acquired using 532 nm laser excitation and a sample temperature of 10 K. The peaks are identified through polarization analysis. Following linearly polarized excitation, the peak at ~ 1700 meV exhibits a degree of linear polarization $(\rho_{CO} - \rho_{CR})/(\rho_{CO}+\rho_{CR}) \approx 15\%$, where ρ_{CO} (ρ_{CR}) is the intensity for co-linearly (cross-linearly) polarized excitation and detection. A linearly polarized peak in photoluminescence can be attributed to the neutral exciton as a consequence of optical pumping of and emission from a coherent superposition of exciton valley states[2]. In contrast, the peak at ~1650 meV does not exhibit valley coherence. The ~50 meV energy separation between the peaks is nearly a factor of two larger than the charged exciton binding energy of ~30 meV relative to the exciton. We therefore attribute this peak to localized excitons, which is also consistent with the WSe_2 photoluminescence peak assignment in Ref. (*2*).

To determine the absorbance of the exciton, defined as $A = 1 - e^{-\alpha L}$, we measured the fractional change in the excitation laser reflectance for a single monolayer flake relative to the

substrate reflectance. The differential reflectance (δ_R) is related to the absorbance of a material on a transparent substrate by[3]

$$\delta_R(\lambda) = \frac{4}{n_s^2 - 1} A \qquad \text{(Supplementary Equation 1)}$$

where $n_s = 1.76$ is the sapphire refractive index. The absorbance is shown in Supplementary Figure 1c for the laser tuned to a similar wavelength used in the nonlinear spectroscopy experiments and a sample temperature of 17 K. From these measurements we find an exciton absorbance $A \approx 0.12$.

Supplementary Note 2

Two-dimensional Fourier-transform spectroscopy is a three-pulse photon echo experiment with the enhancement of interferometric stabilization of the pulse delays. Using a monolithic platform of nested Michelson interferometers with optical delay lines in each path[4], ~100-fs pulses generated from a mode-locked Ti:sapphire laser at a repetition rate of 80 MHz are split into a set of four phase-stabilized pulses. The platform enables femtosecond control of the pulse delays with phase stabilization up to λ/300. Such stability permits Fourier-transformation of the data and allows for phase cycling of the pulse delays to minimize scatter of the excitation pulses into the spectrometer, enhancing the signal-to-noise ratio. Three of the pulses with wavevectors $\boldsymbol{k_A}$, $\boldsymbol{k_B}$, and $\boldsymbol{k_C}$ are focused to a single 35 μm spot FWHM on the sample (Fig. 2a of the main text), which is kept at a temperature of 10 K in a liquid helium cold-finger cryostat. The first pulse, labeled $\boldsymbol{E_A}$ with wavevector $\boldsymbol{k_A}$ in Fig. 2b of the main text, generates an electronic coherence between the crystal ground and exciton states. During the delay τ_A, the individual exciton resonances within the inhomogeneous distribution oscillate out of phase and the macroscopic coherence decays at a rate that is inversely proportional to the inhomogeneous broadening (or in the case of our experiment, the pulse spectral bandwidth). Upon the arrival of field $\boldsymbol{E_A}$ with wavevector $\boldsymbol{k_A}$, the electronic coherences are converted into a transient population grating. After a delay τ_B, field $\boldsymbol{E_C}$ with wavevector $\boldsymbol{k_C}$ generates a coherence whose phase evolution is reverse of that generated by field $\boldsymbol{E_A}$, resulting in a rephasing of the individual frequency components of the inhomogeneously broadened system. The coherent interaction of the three fields with the sample generates a third-order nonlinear optical signal field, $\boldsymbol{E_S}(\tau_A, \tau_B, \tau_C)$, which is a photon echo that is detected in transmission in the wavevector-matching direction $\boldsymbol{k_S} = -\boldsymbol{k_A} + \boldsymbol{k_B} + \boldsymbol{k_C}$. $\boldsymbol{E_S}$ is interferometrically

measured using a fourth phase-stabilized reference field $\boldsymbol{E_R}$ as the delay τ_A (τ_B) is varied for γ (Γ) measurements. Subsequent Fourier transformation of the signal field with respect to τ_C yields a rephasing two-dimensional map of the signal field $\boldsymbol{E_S}$, shown in Supplementary Figure 2. For measurement of γ, we use a value of $\tau_B = 0$ ps to obtain maximum signal-to-noise; however using a value of $\tau_B = 0.2$ ps, which is larger than the pulse autocorrelation duration, does not result in any noticeable difference in the data other than an overall weaker signal strength due to population decay. For each delay τ_A, the pulses are phase cycled to suppress scatter into the phase-matched direction[4]. Phase cycling enhances the signal-to-noise ratio, enabling extraction of the photon echo signal field.

The power of 2DFTS lies in its ability to coherently control the exciton quantum state, enabling unambiguous measurements of exciton dephasing even in the presence of inhomogeneity. To illustrate this we use the Bloch sphere shown in Supplementary Figure 2b, which is a geometrical representation of the dynamical evolution of the exciton quantum mechanical state vector. We show the coherent evolution of the exciton state using $\pi/2$ pulses for clarity; however the experiments are performed in the perturbative regime. Before pulse $\boldsymbol{E_A}$ is incident on the sample, the system is in the crystal ground state $|0\rangle$ at time τ_1. Excitation by pulse $\boldsymbol{E_A}$ at time τ_2 creates a coherent superposition of states $|0\rangle$ and $|1\rangle$ that evolves with frequency ω_i for the ith exciton within the inhomogeneous distribution. As the individual exciton resonances oscillate out of phase, the macroscopic coherence decays by time τ_3. Simultaneous excitation by pulses $\boldsymbol{E_B}$ and $\boldsymbol{E_C}$ at time τ_4 reverses the phase evolution of the individual resonances, resulting in constructive interference and the emission of a photon echo at time $\tau_5 = \tau_A$ after pulse $\boldsymbol{E_C}$.

The photon echo signal field is shown in Supplementary Figure 2b for an exciton excitation density of $N_X = 1.4\times10^{11}$ cm^{-2}. The appearance of the signal along the diagonal dashed line verifies the photon echo formation, which is consistent with the inhomogeneously broadened two-dimensional coherent spectra in Fig. 3 of the main text. We show the time-integrated signal field in Supplementary Figure 2b (points), obtained by projecting the photon echo onto the τ_A axis. The data is fit with a single exponential function (solid line) with decay rate 2γ, which yields the exciton dephasing rate $\gamma = 2.7$ meV (4.0 ± 0.3 ps^{-1}) (see below). The rise time of the signal is due to truncation of the inhomogeneously broadened photon echo signal for $\tau_A \leq 0.1$ ps.

2DFTS offers several advantages compared to other linear and nonlinear spectroscopy techniques. One advantage is the ability to probe both the dephasing rate (γ) by scanning τ_A and the decay rate of the population transient grating (γ_{gr}) by scanning τ_B. In the limit of strong inhomogeneous broadening as observed for the TMD sample, the time-integrated four-wave mixing signal field is[5]

$$\boldsymbol{E_S} \propto \theta(\tau_A)\theta(\tau_B)e^{-2\gamma\tau_A}e^{-\gamma_{gr}\tau_B/2}, \qquad \text{(Supplementary Equation 2)}$$

where θ is the Heaviside Theta function. The transient grating can decay due to both exciton spatial diffusion as well as exciton recombination. Due to strong inhomogeneous broadening, spatial diffusion will also lead to spectral diffusion. We have performed auxiliary two-dimensional coherent spectroscopy experiments (data not shown) and do not observe spectral diffusion on the ultrafast timescales in these measurements and therefore ignore this contribution. Thus, the grating decay rate can be expressed as $\gamma_{gr} = 2\Gamma$ (*5*). The time-integrated photon echo field recorded while scanning τ_B (Fig. 2c of the main text) is therefore a direct measurement of the population recombination rate Γ. Additionally, Supplementary Equation 2 also demonstrates that the time-integrated photon echo signal recorded while scanning τ_A exponentially decays with a rate that is twice the exciton dephasing rate, i.e. 2γ.

To calculate the exciton excitation density, we use the following expression:

$$N_X = \frac{P_{ave}T_p(1-R)\left(1-e^{-\alpha L}\right)}{\pi r^2 E_{ph}}, \qquad \text{(Supplementary Equation 3)}$$

where P_{ave} is the average power per beam, $T_p = 12.5$ ns is the laser pulse time separation, $R = 0.15$ takes into account reflection losses, $A = 1 - e^{-\alpha L} = 0.12$ is the linear absorbance of the WSe_2 monolayer, $r = 17.5$ μm is the focused beam radius, and $E_{ph} = 1710$ meV is the photon energy. For all excitation densities used in the experiments, the signal field amplitude exhibits a $\chi^{(3)}$ behavior superimposed on a weak linear background term due to incomplete suppression of scattered light in the phase-matched direction. The laser excitation spot size for both the photoluminescence and nonlinear spectroscopy experiments is larger than the typical monolayer flake size. We have obtained similar spectroscopy results in the text for several locations on the sample, demonstrating the uniformity in the optical properties. Excitons localized at the monolayer flake edges exhibit a

spectral shift to longer wavelength, which is observed in our sample as well as in other experiments[6,7]. Because the excitation laser is tuned to the higher energy side of the exciton resonance in the 2DFTS experiments and the number of excitons localized at the edge states is small compared to the flake center, we speculate that edge states have minimal impact on the coherent dynamics measured in this work. We have also measured a similar dephasing rate of γ= 2.4 meV from a different CVD-grown WSe_2 monolayer sample exhibiting reduced photoluminescence from defect-bound excitons for an excitation density of N_X = 1.3×10^{11} cm^{-2} (Supplementary Figure 6).

Supplementary Note 3

Starting with Maxwell equations and solving the wave equation for two-dimensional TMDs, we calculate the frequency-dependent excitonic absorbance $\alpha(\omega) = 1 - T(\omega) - R(\omega)$ with the transmission $T(\omega)$ and the reflection coefficient $R(\omega)$. Exploiting the boundary conditions for the electrical field for a TMD monolayer located between two media characterized by the refractive indices n_1 and n_2, we obtain the following analytic expression for the absorbance[8-10]

$$\alpha(\omega) = \frac{\frac{\omega}{c_0 n_1} Im[\chi_{2D}(\omega)]}{|\frac{1}{2}\left(1+\frac{n_1}{n_2}\right) - i\frac{\omega}{2c_0 n_1}\chi_{2D}(\omega)|^2} \quad \text{(Supplementary Equation 4)}$$

with the speed of light c_0 in vacuum and the optical susceptibility $\chi_{2D}(\omega)$ describing the linear response of the TMD monolayer to an optical pulse. To obtain this material-specific quantity we evaluate semiconductor Bloch equations resulting in the expression[8]

$$\chi_{2D}(\omega) = \frac{1}{\varepsilon_0 \omega^2} \sum_{\nu\xi s} \frac{\Theta_{\nu\xi}^{s}}{E_{\nu\xi}^{s} - \hbar\omega - i\gamma_s} \quad \text{(Supplementary Equation 5)}$$

corresponding to the Elliott formula including the electrical permittivity ε_0 and a small parameter γ_s that is necessary for numerical reasons and that has no influence on the calculated radiative life time. The optical susceptibility $\chi_{2D}(\omega)$ is determined by the excitonic eigenfunctions $\Theta_{\nu\xi}^{s}$ (here also including the optical matrix element) and eigenvalues $E_{\nu\xi}^{s}$ of the Wannier equation. Here, ν, ξ, s are the indices describing the excitonic state, the valley, and the spin, respectively. Supplementary Figure 4 shows the absorbance $\alpha(\omega)$ focusing on the A exciton for monolayer WSe_2 on a sapphire substrate (n_1 = 1.75). Our calculations reveal a homogeneous linewidth of 1.43

meV corresponding to a radiative lifetime of 230 fs. This value is consistent with the measurements and provides a lower bound on the exciton radiative lifetime.

The calculated radiative coupling depends on the refractive index of the substrate, the strength of the excitonic absorption, and the intrinsic material parameters, such as the effective mass. The calculation has been performed for monolayer WSe_2 grown on sapphire substrate (n_1 = 1.75, n_2 = 1 corresponding to vacuum). We find that the higher the substrate refractive index n_1 resulting in a more efficient screening of the Coulomb interaction, the longer is the radiative lifetime. In Supplementary Figure 5, the radiative linewidth of the four most prominent TMD materials including WSe_2,WS_2, MoS_2, and $MoSe_2$ is shown as a function of the dielectric environment (n_1). The strongest Coulomb interaction can be found for the free standing TMDs with n_1 = 1. In this case, we find a linewidth of 3.6 meV for WSe_2 corresponding to a radiative lifetime of about 90 fs. Furthermore, the excitonic oscillator strength is given by $\Theta_{\nu\xi}^{s}$ that contains both the optical matrix element and the excitonic wave function[11]. For all TMD materials, the strength of the carrier-light interaction has been adjusted to the experimentally measured absorption of ~10% at the *A* exciton for WSe_2. Doubling the absorption to 20% increases the linewidth from γ = 1.43 meV to γ = 2.85 meV for WSe_2 on sapphire, i.e. the more efficient the absorption, the larger is the radiative broadening and the shorter is the radiative life time.

Finally, our calculations show that TMD monolayers with a higher effective mass (entering the excitonic wave function) exhibit a stronger radiative coupling. This can be seen in Supplementary Figure 5 where the members of the molybdenum family having a higher effective mass[12], exhibit a stronger coupling. This can be traced back to an enhanced excitonic wave function for heavier holes and electrons. Another important factor is the spectral position of the excitonic resonance, which enters into the equation via the excitation frequency ω. The higher ω, the shorter is the radiative lifetime. Considering all mentioned dependencies, our calculations reveal the shortest radiative lifetime of approximately 60 fs for MoS_2 in the free-standing case, cf. Supplementary Figure 5.

Supplementary References

1. Huang, J.-K. *et al.*, Large-area synthesis of highly crystalline WSe_2 monolayers and device applications, *ACS Nano* **8,** 923–930 (2013).

2. Wang, G. *et al.*, Giant enhancement of the optical second-harmonic emission of WSe_2 monolayers by laser excitation at exciton resonances, *Phys Rev. Lett.* **114**, 097403 (2015).

3. Mak, K. F., Lee, C., Hone, J., Shan, J. & Heinz, T. F., Atomically thin MoS_2: a new direct-gap semiconductor, *Phys. Rev. Lett.* **105,** 136805 (2010).

4. Bristow, A. D. *et al.*, A versatile ultra-stable platform for optical multidimensional Fourier-transform spectroscopy, *Rev. Sci. Instrum.* **80**, 073108 (2009).

5. Cundiff, S. T., Coherent optical spectroscopy of semiconductors, *Opt. Express* **16**, 4639-4664 (2008).

6. Gutierrez, H. R. *et al.*, Extraordinary room-temperature photoluminescence in triangular WS_2 monolayers, *Nano Lett.* **13**, 3447-3454 (2013).

7. Yin, X. *et al.*, Edge nonlinear optics on a MoS_2 atomic monolayer, *Science* **344**, 488-490 (2014).

8. Stroucken, T., Knorr, A., Thomas, P., and Koch, S., Coherent dynamics of radiatively coupled quantum-well excitons, *Phys. Rev. B* **53**, 2026 (1996).

9. Stauber, T., Peres, N., and Geim, A., Optical conductivity of graphene in the visible region of the spectrum, *Phys. Rev. B* **78**, 85432 (2008).

10. Malic, E. and Knorr, A., *Graphene and Carbon Nanotubes: Ultrafast Optics and Relaxation Dynamics* (Wiley-VCH, Berlin, 2013).

11. Berghäuser, G. and Malic, E., Analytical approach to excitonic properties of MoS_2, *Phys. Rev. B* **89**, 125309 (2014).

12. A. Ramasubramaniam, Large excitonic effects in monolayers of molybdenum and tungsten dichalcogenides, *Phys. Rev. B* **86**, 115409 (2012).
